# Design of experiments and the virtual PCR simulator: An online game for pharmaceutical scientists and biotechnologists

**DOI:** 10.1002/pst.1932

**Published:** 2019-02-21

**Authors:** Harold Fellermann, Ben Shirt‐Ediss, Jerzy Kozyra, Matt Linsley, Dennis Lendrem, John Isaacs, Thomas Howard

**Affiliations:** ^1^ Interdisciplinary Computing and Complex Biosystems Research Group, School of Computing Science Newcastle University Newcastle upon Tyne UK; ^2^ School of Computing Newcastle University Newcastle upon Tyne UK; ^3^ Mathematics and Statistics Newcastle University Newcastle upon Tyne UK; ^4^ National Institute for Health Research, Newcastle Biomedical Research Centre, Newcastle upon Tyne Hospitals NHS Foundation Trust and Newcastle University, Institute of Cellular Medicine (Musculoskeletal Research Group) Newcastle upon Tyne UK; ^5^ Newcastle University, Institute of Cellular Medicine, Musculoskeletal Research Group, Newcastle University and Newcastle upon Tyne Hospitals NHS Foundation Trust Newcastle upon Tyne UK; ^6^ School of Natural and Environmental Sciences Newcastle University Newcastle upon Tyne UK

Design of experiments (DOE) enables scientists to explore complex, multidimensional spaces against a background of experimental variability with the minimum of resource. While these methods are most successful when combined with expert knowledge to define the design space and capture the dimensionality of the problem, many scientists believe, often quite strongly, that they can deliver insights into these complex, multidimensional spaces without recourse to DOE tools. Many believe that small experiments, guided by scientific intuition alone, exploring the changes as each variable in turn is changed, one factor at a time (OFAT), are more efficient than DOE. This belief is strong and persists, even in the face of data demonstrating that it is clearly wrong.^1^

This situation is perpetuated by scientific teaching in support of OFAT approaches to scientific experimentation. Although OFAT methods have been largely discredited, their use in pharmaceutical R&D persists partly because OFAT is taught in schools as part of the “scientific method,” partly because of the illusion that it is an efficient methodology and partly because OFAT methods have the beguiling property of generating data confirming initial scientific beliefs. Many simple ridge systems or valley systems will generate data to confirm the initial starting points are correct even when seriously flawed—the *Paradox of the Self‐Fulfilling Prophecy*.^2^

Simulation can be a useful teaching tool.^3^, ^4^, ^5^, ^6^ For this reason, we developed a web‐based application designed to introduce concepts of multifactorial experimental design and support teaching of the polymerase chain reaction—the virtual PCR simulator. Learners select experimental settings and receive results of their simulated reactions quickly, allowing rapid iteration between data generation and analysis. This enables the student to perform complex iterative experimental design strategies within a short teaching session. Here, we provide a brief overview of the user interface and describe our experience using this tool in a teaching environment.

## 1 THE VIRTUAL PCR SIMULATOR

Described in full elsewhere, the simulator is available on the Newcastle University server at http://virtual‐pcr.ico2s.org/ where it records design decisions, experiments, and results for all experiments.^7^ Students may select settings for 12 experimental factors—see Table 1. DNA amplification through PCR involves cycling through three key steps—denaturation, annealing, and extension. The temperature and duration of each these three steps are controlled during each cycle. In addition, there are four reagent volumes and one categorical choice of polymerase.

**Table 1:** Experimental factors and permitted working ranges for the virtual PCR simulator

Factor	Lower Boundary	Upper Boundary
Number of cycles	1	50
Denaturation temperature	1	100
Denaturation time	1	‐‐‐
Annealing temperature	1	100
Annealing time	1	‐‐‐
Extension temperature	1	100
Extension time	1	‐‐‐
dNTP concentration	0	20
Primer concentration	0	20
Plasmid mass	0	20
Polymerase concentration	0	20
Polymerase type	Phusion or Taq polymerase	

The objective is to maximize yield and purity of the amplified DNA while minimizing the total cycle time. Users perform experiments. The results are then used to create a leader board where users are ranked over a gliding window of 1 hour. Gaming introduces a competitive element and is an effective motivational tool.^4^, ^6^

PCR experiments are simulated by numerically integrating a differential equation model of the reaction, subject to the selected temperature cycle and initial reagent concentrations. The concentrations of the latter are subjected to pipetting “noise” using random values within acceptable operating boundaries of commonly used pipettes. The model captures melting and annealing of all DNA species, binding and unbinding of the polymerase, primer extension, and polymerase degradation. Rates of all reactions are dependent on the temperature.^7^ The model qualitatively captures parameter responses of PCR while producing numerical results within seconds.

**Figure 1:**
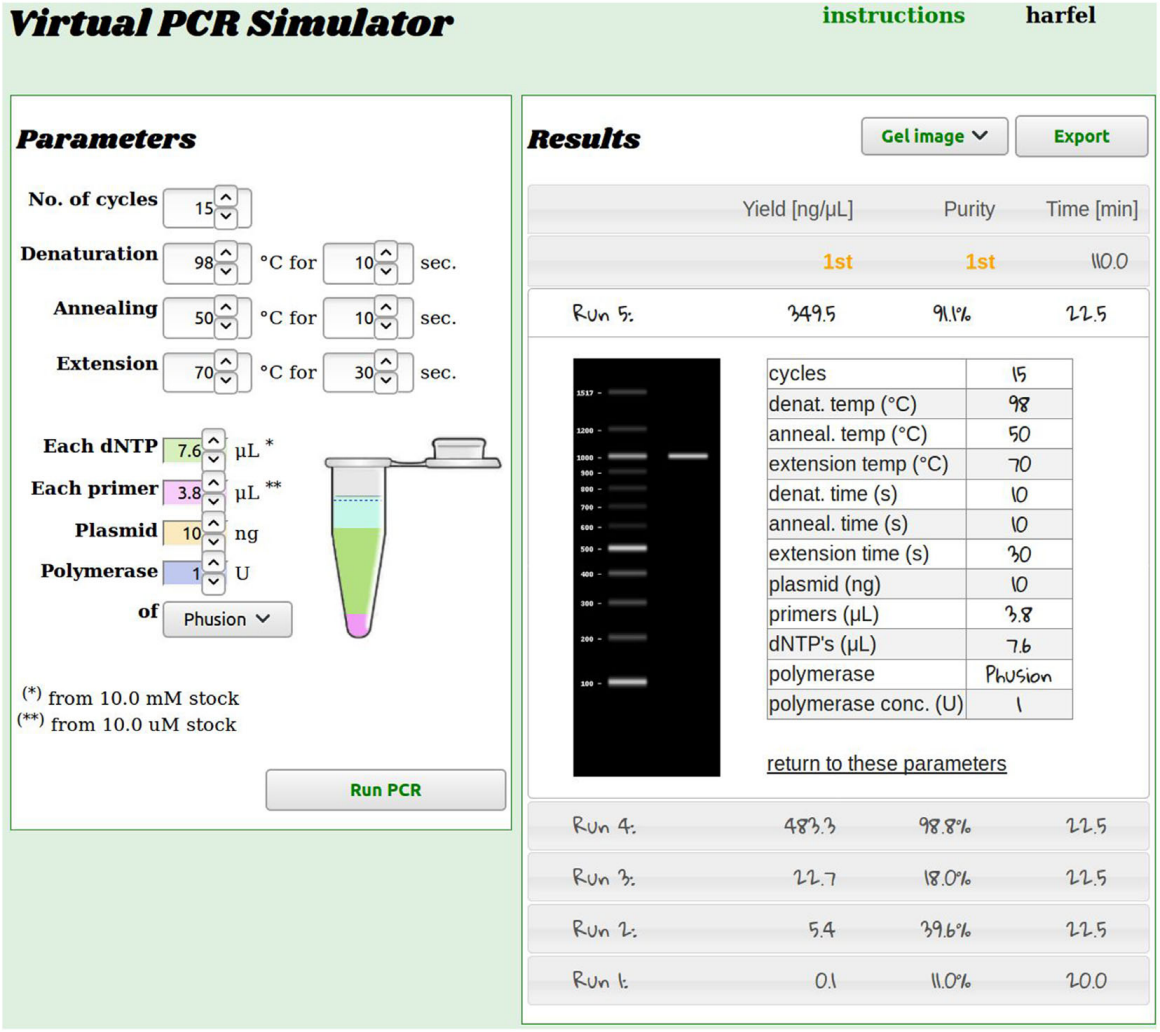
The PCR simulator allows scientists to run cloning experiments for a range of conditions for a gene amplification polymer chain reaction. The goal is to maximize yields and purity while minimizing cycle time. The user is free to choose from a range of conditions. The server simulates the results, generates a gel image, and records the data in a virtual “laboratory notebook.” See text for details

The user interface is a single web page, or “laboratory notebook,” combining an input form for experimental settings, data from all experiments with simulated electrophoresis gel images, and a parameter table from which the user can return to any previously used settings (see Figure 1). The “lab book” also displays the rank of the user's performance within their cohort. Experiments (factors and responses) can be downloaded in CSV format for offline analysis and experimental planning. When the simulator is in qPCR mode, the user may toggle between gel images and DNA concentration time series.

## 2 THE VIRTUAL PCR SIMULATOR AS A TEACHING TOOL

For the last 3 years, we have employed the simulator to teach DoE to small cohorts of around 20 scientists drawn from academic and industrial backgrounds with a range of laboratory and statistical experience. In each class, the simulator sessions are interspersed with lectures, seminars, and workshop activities and last 15 to 45 minutes. Participants apply learning from various sessions in their interactions with the simulator, starting with free exploration of parameter space moving towards screening and optimization experiments. The competitive leader‐board component enhances engagement. Data can be exported and interrogated providing information to the instructors and feedback to the scientist.

**Figure 2:**
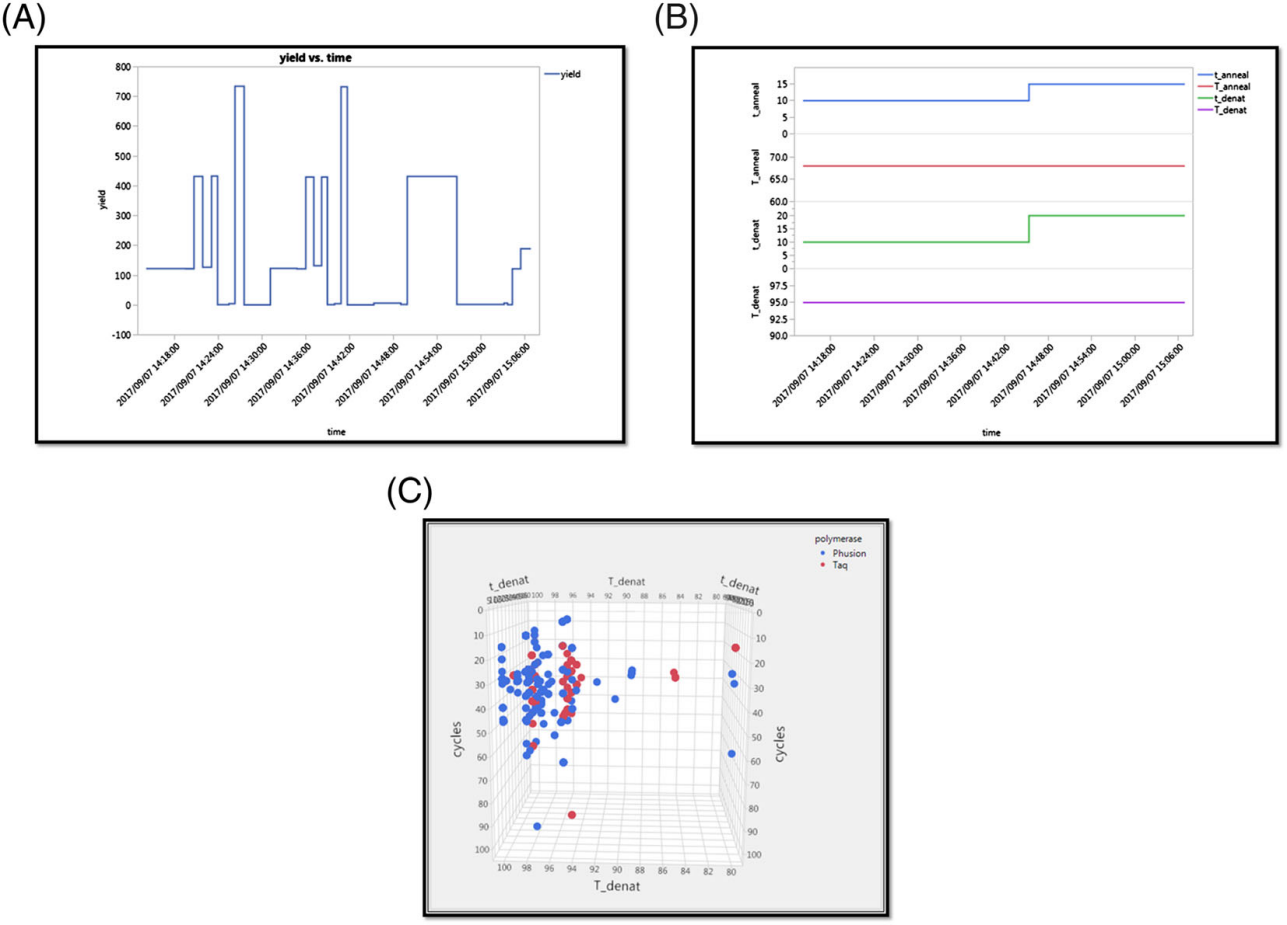
Real‐time data can be extracted from the PCR simulator allowing the class to monitor their progress. In panel A, we have data for an individual scientist performing experiments, one at a time over a 40‐minute practical. The yield of their process is plotted together with their choices of settings for four of the parameters—denaturing time, denaturing temperature, annealing time, and annealing temperature (panel B). Note that denaturing and annealing temperatures are not tested at all, and much of the design space remains unexplored. This is not an isolated example. Even large groups of highly experienced scientists, free to explore the design space however they want, leave much of the design space completely unexplored. In panel C, we have plotted three of the variables—the number of cycles, together with the denaturing temperature and denaturing time, for Phusion and Taq polymerases—for an entire class. Most experiments are performed in narrow regions of the design space, some variables are not investigated at all, and their impact upon robustness is completely untested. See text for details

The PCR simulator provides a virtual experience of a complex laboratory protocol with the simulated reactions qualitatively matching real experiments. We have used it to assess user engagement with the task and to demonstrate how traditional approaches to experimentation often result in suboptimal results, narrow search strategies, and a poor understanding of variability and reproducibility (Figure 2). The teaching interface allows the instructor to draw out personal and group learning. This includes information on the performance of individual users, and how widely or effectively the cohort is exploring potential experimental options.

## 3 CONCLUSION

Our experience has been that the virtual PCR simulator shatters, once and for all, the delusion that the complex spaces we are asked to explore in pharmaceutical R&D and biotechnology are amenable to simplistic OFAT approaches. The problems we work on simply do not lend themselves to such naïve experimentation. Attendees tell us that the simulator demonstrates the real value of a statistical, designed approach. The virtual PCR simulator allows scientists to explore these complex multidimensional spaces in the comparative safety of a simulation environment before trying the tools in the laboratory or the plant.

We invite others to explore the tool at http://virtual‐pcr.ico2s.org/.

## AUTHOR CONTRIBUTIONS

H.F., B.S.E., and J.W.K. wrote the software and mathematical model. T.P.H. advised on principles and applications of PCR. H.F., M.L., D.L., and T.P.H. tested and iterated the simulator in a teaching environment. All authors contributed to writing the manuscript.

## DATA AVAILABILITY STATEMENT

The data that support the findings of this study are openly available in **bioRxiv** at https://doi.org/10.1101/415042.
